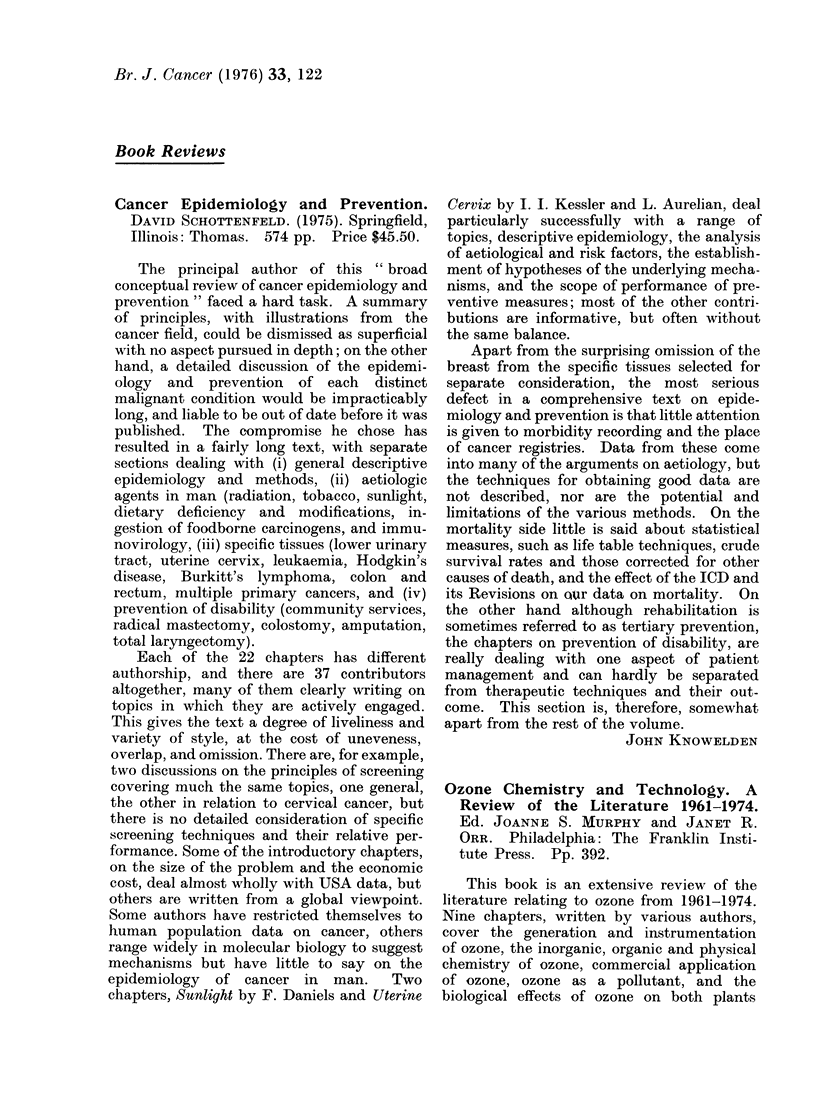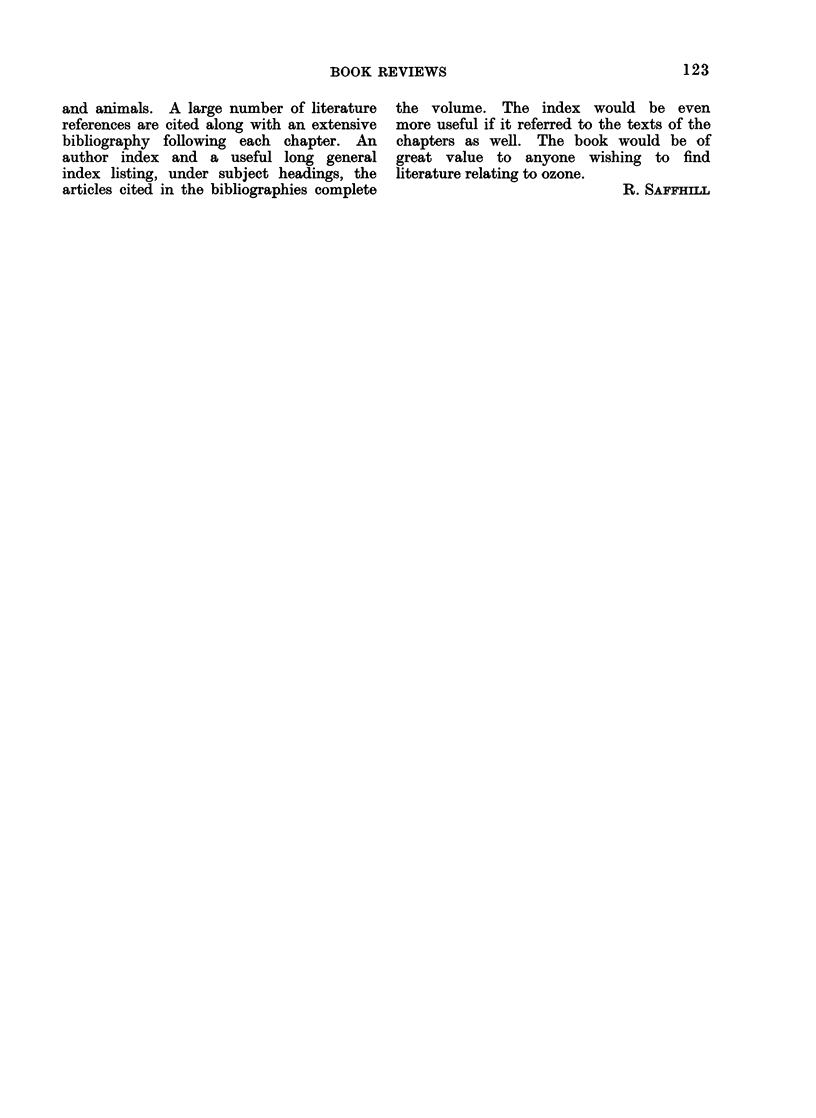# Ozone Chemistry and Technology. A Review of the Literature 1961-1974

**Published:** 1976-01

**Authors:** R. Saffhill


**Ozone Chemistry and Technology. A Review of the Literature 1961–1974.** Ed. JOANNE S. MURPHY and JANET R. ORR. Philadelphia: The Franklin Institute Press. Pp. 392.

This book is an extensive review of the literature relating to ozone from 1961–1974. Nine chapters, written by various authors, cover the generation and instrumentation of ozone, the inorganic, organic and physical chemistry of ozone, commercial application of ozone, ozone as a pollutant, and the biological effects of ozone on both plants and animals. A large number of literature references are cited along with an extensive bibliography following each chapter. An author index and a useful long general index listing, under subject headings, the articles cited in the bibliographies complete the volume. The index would be even more useful if it referred to the texts of the chapters as well. The book would be of great value to anyone wishing to find literature relating to ozone.

R. SAFFHILL